# Risk-adapted first-stage patent ductus arteriosus ligation for an anomalous left brachiocephalic trunk arising from the main pulmonary artery in a critically ill neonate: a case report and literature review

**DOI:** 10.1097/RC9.0000000000001111

**Published:** 2026-10-14

**Authors:** Kittipos Peerapatanapong, Piya Samankatiwat

**Affiliations:** Division of Cardiothoracic Surgery, Department of Surgery, Faculty of Medicine, Ramathibodi Hospital, Mahidol University, Bangkok, Thailand

**Keywords:** anomalous brachiocephalic trunk, case report, literature review, neonatal cardiac surgery, patent ductus arteriosus, right-sided aortic arch

## Abstract

**Introduction and importance:**

Anomalous origin of the brachiocephalic trunk from the main pulmonary artery (MPA) is an exceptionally rare congenital vascular anomaly. Management is not standardized, particularly in critically ill neonates.

**Case presentation:**

A female neonate developed acute respiratory distress after birth. Echocardiography showed an atrial septal defect and a patent ductus arteriosus (PDA). Follow-up echocardiography and computed tomography angiography confirmed a right-sided aortic arch, a right-sided PDA, and an anomalous left brachiocephalic trunk arising from the MPA. The course was complicated by active infection, mechanical ventilation, liver cirrhosis, thrombocytopenia, and coagulopathy. After multidisciplinary review, PDA ligation alone was selected as a first-stage stabilization strategy to eliminate the dominant left-to-right shunt. Median sternotomy allowed exposure of the right-sided PDA and great vessels. The PDA was ligated without cardiopulmonary bypass, and the anomalous vessel was preserved. Complete PDA closure and temporary hemodynamic improvement were observed. However, after transfer back to the referring hospital, the patient died before second-stage reconstruction could be performed.

**Clinical discussion:**

Reported strategies include vessel ligation and single-stage anatomical repair. In this neonate, definitive reconstruction was deferred because of severe systemic comorbidities and poor physiological reserve. PDA ligation may provide temporary hemodynamic stabilization in critically ill neonates; however, it did not correct the anomalous vascular anatomy or establish long-term perfusion of the cerebral circulation and the left upper extremity.

**Conclusion:**

Treatment should be individualized according to physiological reserve. PDA ligation alone may be considered as a temporary, risk-adapted first-stage stabilization in selected high-risk neonates when complete correction is unsafe.

## Introduction

Congenital anomalies of the aortic arch and its branches are uncommon. The anomalous origin of the brachiocephalic trunk from the main pulmonary artery (MPA) is exceptionally rare. Since the first description by Fong and Venables^[^1^]^, only a few related cases have been reported^[^2,3^]^. Owing to the limited literature, management remains uncertain, particularly in critically ill neonates. We report a neonate with an anomalous left brachiocephalic trunk arising from the MPA, a right-sided aortic arch, and a right-sided patent ductus arteriosus (PDA), in whom PDA ligation alone was performed as a temporary first-stage stabilization measure, with short-term hemodynamic improvement. We also review similar reported cases and discuss the rationale and limitations of this risk-adapted approach. This case has been reported in line with the 2025 SCARE criteria^[^4^]^.

HIGHLIGHTS

- A rare anomalous left brachiocephalic trunk arose from the main pulmonary artery.
- A hemodynamically significant patent ductus arteriosus (PDA) contributed to pulmonary over circulation, systemic hypoperfusion, and congestive heart failure.
- Staged management may be considered in selected neonates with multiple systemic comorbidities.
- Median sternotomy enabled direct exposure of complex right-sided ductal anatomy.
- Off-pump PDA ligation achieved early hemodynamic stabilization.

## Presentation of case

A female neonate developed acute respiratory distress at 31 hours after birth. Echocardiography demonstrated an atrial septal defect and a PDA with left-to-right shunting. The clinical course was complicated by recurrent infection, sepsis, cholestatic jaundice, and liver cirrhosis. Follow-up echocardiography on day-of-life 25 showed progressive PDA enlargement and a right-sided aortic arch, raising suspicion of an associated vascular anomaly. Computed tomography angiography subsequently demonstrated a right-sided aortic arch, an anomalous left brachiocephalic trunk arising from the MPA and bifurcating into the left common carotid and left subclavian arteries, and a 7 mm right-sided PDA connecting the aortic isthmus to the right pulmonary artery (Figs. 1 and 2).

**Figure 1.**
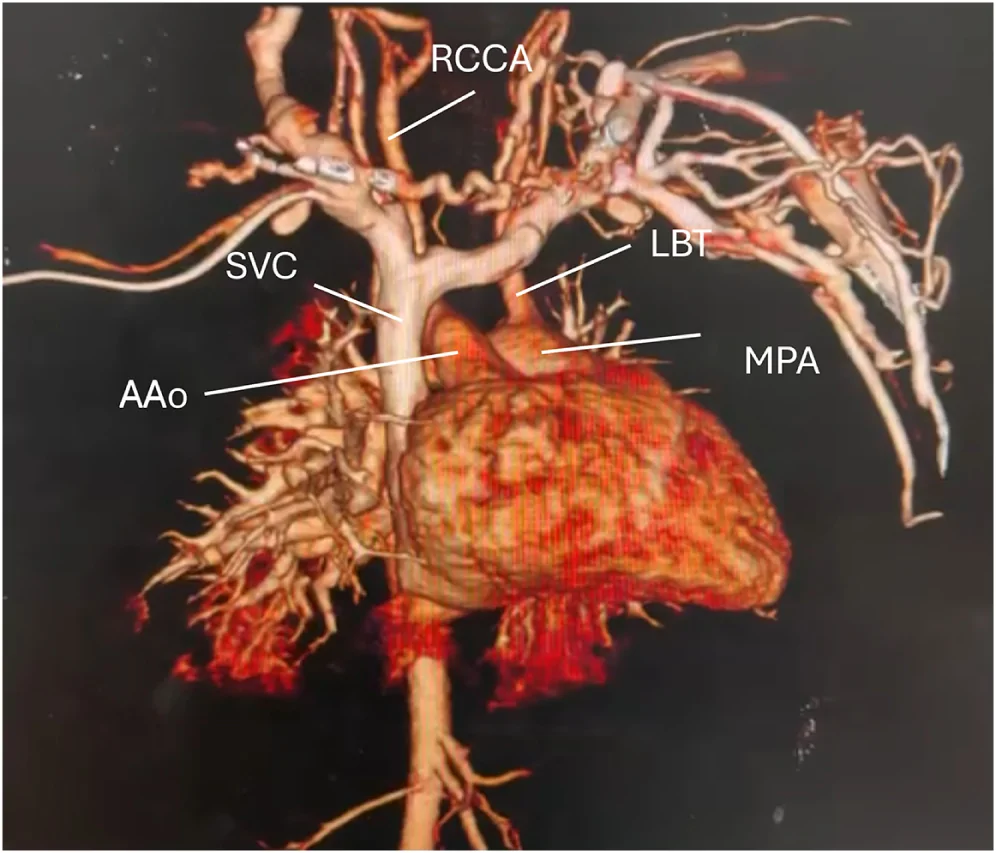
Three-dimensional computed tomography angiography demonstrating an anomalous left brachiocephalic trunk (LBT) arising from the main pulmonary artery (MPA), with the ascending aorta (AAo), right common carotid artery (RCCA), and superior vena cava (SVC) shown for anatomical orientation.

**Figure 2.**
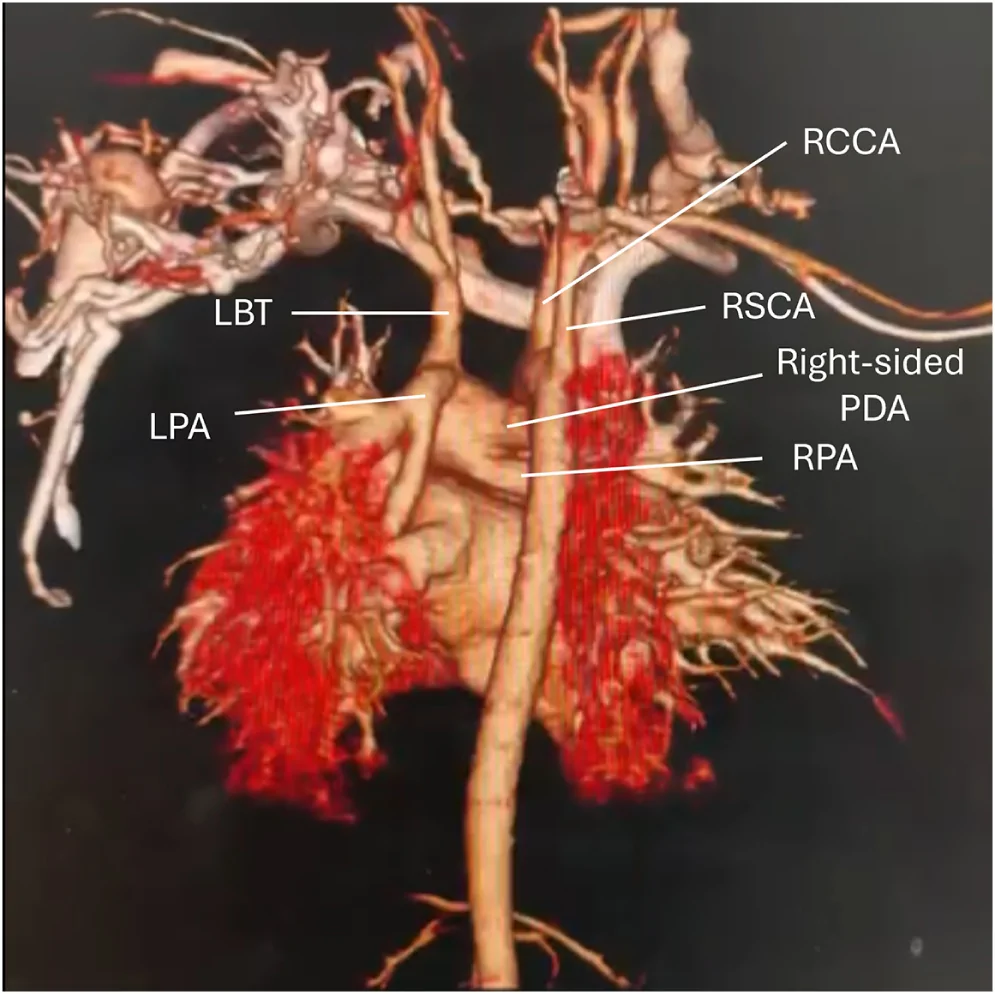
Three-dimensional computed tomography angiography demonstrating the right-sided patent ductus arteriosus (PDA) in relation to the right pulmonary artery (RPA), left pulmonary artery (LPA), right common carotid artery (RCCA), right subclavian artery (RSCA), and anomalous left brachiocephalic trunk (LBT).

She was referred to a tertiary cardiac surgical center at approximately 1 month of age. At the time of referral, she remained mechanically ventilated. Her clinical course was complicated by congestive heart failure, recurrent pneumonia, necrotizing enterocolitis, and liver cirrhosis. Preoperative management included mechanical ventilatory support, dobutamine infusion at 10 μg/kg/min, intravenous diuretic therapy, broad-spectrum intravenous antibiotics, including meropenem and colistin, and correction of coagulopathy and thrombocytopenia with blood components. No known drug allergies were documented. There was no relevant family history of congenital heart disease.

A multidisciplinary team considered three strategies: staged PDA ligation alone, PDA ligation with simultaneous reimplantation of the anomalous vessel, and complete anatomical correction. Because of active infection, necrotizing enterocolitis, hepatic dysfunction, thrombocytopenia, coagulopathy, and overall clinical fragility, immediate complete correction was deferred. The large right-sided PDA, with substantial left-to-right shunting, was considered a major contributor to congestive heart failure and cardiopulmonary instability in the setting of recurrent pneumonia and necrotizing enterocolitis. Therefore, the initial operation focused on PDA ligation as a first-stage stabilization procedure.

The operation was performed by a pediatric cardiothoracic surgeon and a chief resident. The patient was under general anesthesia in the supine position. Median sternotomy was selected rather than thoracotomy because of the right-sided ductal anatomy and the need for direct exposure of the great vessels. Intraoperative findings confirmed the right-sided aortic arch, a 7 mm PDA, and an anomalous left brachiocephalic trunk arising from the anterior surface of the MPA. The PDA was ligated without cardiopulmonary bypass, and the anomalous vessel was preserved. The operation proceeded as planned.

Postoperative echocardiography confirmed complete PDA closure without residual ductal flow. Short-term clinical signs of congestive heart failure improved after PDA ligation, with approximately a 10 mm Hg increase in diastolic blood pressure, although dobutamine infusion, intravenous diuretic therapy, and mechanical ventilatory support were continued because of ongoing pneumonia, necrotizing enterocolitis, and systemic illness. On physical examination, there was no clinically significant difference in blood pressure or oxygen saturation between the two upper extremities. No clinically evident early neurological deficit was observed.

The patient was transferred back to the referring hospital approximately 1 week after surgery for continued management of heart failure, infection, necrotizing enterocolitis, and hepatic dysfunction. After being transferred back, the patient developed recurrent infection-related clinical deterioration. After discussion with the treating team, the family elected not to pursue further resuscitative measures. The patient subsequently died before second-stage anatomical reconstruction could be performed.

## Discussion

The embryological basis of this anomaly remains incompletely understood. The double-aortic arch model described by Edwards explains many arch anomalies but does not fully account for a brachiocephalic branch arising from the pulmonary artery^[^5^]^. Abnormal aortopulmonary septation and neural crest migration have been proposed as possible mechanisms^[^6,7^]^.

Right-sided PDA is an uncommon ductal laterality variant, usually associated with a right-sided aortic arch. Right-sided aortic arch occurs in approximately 0.1% of the general population, and a right-sided arterial duct has been reported in approximately 10%–15% of patients with a right-sided aortic arch^[^8^].^ Its clinical relevance lies in diagnostic complexity, altered surgical exposure, and the need to define the complete ductal and supra-aortic branch anatomy before intervention.

Only a few cases of carotid or brachiocephalic arterial origin from the pulmonary artery have been reported (Table 1). Fong and Venables^[^1^]^ reported two infants with isolated left innominate or left common carotid arterial anatomy associated with ductal or pulmonary arterial connections. Osakwe *et al*^[^2^]^ described an anomalous origin of the left common carotid artery from the MPA, which was managed by ligation after confirmation of adequate collateral cerebral circulation. Tran *et al*^[^3^]^ reported a similar anomalous left brachiocephalic trunk associated with a right-sided aortic arch and a right-sided PDA, managed with single-stage repair in a patient without major systemic comorbidities. These reports suggest that management strategies are not uniform and should be individualized according to cerebral and upper-extremity perfusion, anatomical feasibility, and physiological reserve.

**Table 1:** Previously reported cases of isolated carotid or brachiocephalic arterial anatomy with pulmonary arterial or ductal connection.

Author/year	Age	Anomalous arterial anatomy	Associated anomalies	Clinical/perfusion findings	Management	Outcome/follow-up
Fong and Venables (1987)^[^1^]^	6 weeks	Isolated LCCA connected to the MPA by LDA.	RAA.	Clinical: tachypnea	Left ductus arteriosus ligation had been performed.	Sudden death before the second stage could be performed.
			CHARGE syndrome.			
				Perfusion: not done, but there was a note of a complete circle of Willis in the necropsy.		
Patient 1						
					Further surgery was planned.	
Patient 2	10 months	Isolated LCCA connected to the MPA through a ductus arteriosus.	RAA	Clinical: not mentioned	Ligated LCCA with ASD closure and channeling drainage of persistent left superior vena cava to right atrium.	Uneventful postoperative course.
			Ostium primum ASD.			
				Perfusion: retrograde filling of the left common carotid artery to the pulmonary artery from the circle of Willis.		
						No long-term follow-up.
			Persistent left superior vena cava.			
		Aberrant LSCA arising from the lower descending aorta.				
			Dwarfism			
Osakwe *et al* (2016)^[^2^]^	Neonate	Anomalous LCCA arising from the MPA.	CHARGE syndrome.	Clinical: tachypnea	ASD and VSD closure with ligation and release of anomalous LCCA from its origin.	Uneventful postoperative course.
				Perfusion: retrograde LCCA flow created cerebral steal; persistent trigeminal artery provided collateral cerebral circulation.		
		Aberrant LSCA.				
			RAA.			
			Ebstein’s anomaly.			
		Persistent left trigeminal artery				
						No long-term follow-up.
			VSD and ASD.			
			Bicuspid aortic valve			
Tran *et al* (2020)^[^3^]^	3 weeks	Anomalous left innominate/brachiocephalic trunk arising from the pulmonary trunk.	Right-sided aortic arch	Clinical: cyanosis while feeding.	Single-stage anatomical repair.	Successful surgical management; hearing loss was noted during postoperative follow-up.
				Perfusion: no preoperative study was performed.		
			Right-sided PDA			
			bilateral choanal atresia			
Present case	1 month	Anomalous LBT arising from the MPA and bifurcating into the LCCA and LSCA.	ASD.	Clinical: critically ill neonate with heart failure, ventilator dependence, recurrent infection/sepsis, pneumonia, necrotizing enterocolitis, liver cirrhosis, thrombocytopenia, and coagulopathy.	First-stage off-pump PDA ligation; anomalous vessel preserved.	Complete PDA closure and early hemodynamic stabilization; died from recurrent infection-related deterioration before the second stage could be performed.
		RAA.				
					Definitive anatomical reconstruction was planned but could not be performed.	
				Perfusion: no preoperative study was performed.		

ASD, atrial septal defect; VSD, ventricular septal defect; RAA, right-sided aortic arch; LBT, left brachiocephalic trunk; LCCA, left common carotid artery; LSCA, left subclavian artery; MPA, main pulmonary artery; PDA, patent ductus arteriosus; LDA, left ductus arteriosus.

Data are summarized from published reports when available; when operative or follow-up details are not clearly reported, this is stated explicitly.

In a clinically stable patient, complete anatomical correction is generally considered the definitive treatment because it eliminates left-to-right shunting and restores systemic arterial perfusion to the left carotid and subclavian circulation. In the present case, however, the large right-sided PDA was considered the dominant contributor to pulmonary overcirculation, low diastolic pressure, systemic hypoperfusion, and congestive heart failure^[^9^]^. Therefore, ductal ligation was expected to reduce the immediate shunt burden and provide short-term improvement.

Immediate definitive reconstruction was deferred because it would have required complex neonatal vascular reconstruction and might have required cardiopulmonary bypass if intracardiac repair was undertaken. Cardiopulmonary bypass in patients with significant hepatic dysfunction or cirrhosis has been associated with increased perioperative morbidity and mortality^[^10^]^. Together with the patient’s severe systemic comorbidities and overall clinical fragility, these factors supported PDA ligation alone as an individualized first-stage stabilization procedure while preserving the anomalous vessel and deferring definitive repair.

However, the major limitation of this staged strategy was the persistence of the anomalous left brachiocephalic trunk arising from the pulmonary artery. This persistent anatomy left ongoing concerns regarding cerebral and left upper-extremity perfusion, including low-pressure pulmonary arterial supply, pulmonary steal, and thromboembolic risk. Although no clinically significant inter-arm differences in blood pressure or oxygen saturation and no clinically evident early neurological deficit were observed before transfer, these were only short-term bedside findings and could not establish the long-term adequacy of cerebral and left upper-extremity perfusion, vessel growth, or neurodevelopmental outcome. These outcomes could not be assessed because the patient died of recurrent infection-related clinical deterioration after being transferred back, before the second-stage could be performed.

The main strength of this report is its focus on operative decision-making for an exceptionally rare anomaly with major systemic comorbidities. The principal limitations are the single-case design, the absence of second-stage anatomical reconstruction, and the lack of long-term data on cerebral and left upper extremity perfusion, vessel growth, and neurodevelopmental outcomes.

## Conclusion

Anomalous origin of the left brachiocephalic trunk from the MPA with right-sided PDA is exceptionally rare. In critically ill neonates with poor physiological reserve, PDA ligation alone may be considered for short-term hemodynamic stabilization while complete anatomical correction is unsafe. However, it does not correct the underlying anomalous vascular anatomy. Long-term cerebral and left upper-extremity perfusion, vessel growth, and neurodevelopmental outcomes remain unresolved. This case emphasizes the importance of individualized surgical planning based on anatomy, hemodynamic priority, and physiological reserve.

## Data Availability

The data supporting the findings of this case report are available from the corresponding author upon reasonable request.
